# ^177^Lu-DOTATATE therapy in metastatic/inoperable pheochromocytoma-paraganglioma

**DOI:** 10.1530/EC-20-0292

**Published:** 2020-08-12

**Authors:** Sanjeet Kumar Jaiswal, Vijaya Sarathi, Saba Samad Memon, Robin Garg, Gaurav Malhotra, Priyanka Verma, Ravikumar Shah, Manjeet Kaur Sehemby, Virendra A Patil, Swati Jadhav, Anurag Ranjan Lila, Nalini S Shah, Tushar R Bandgar

**Affiliations:** 1 Department of Endocrinology, Seth G.S Medical College and KEM Hospital, Mumbai, Maharashtra, India; 2 Department of Endocrinology, Vydehi Institute of Medical Sciences and Research Center, Bengaluru, Karnataka, India; 3 Department of Nuclear Medicine, Bhabha Atomic Research Centre, Mumbai, Maharashtra, India

**Keywords:** pheochromocytoma, paraganglioma, PRRT, predictors of response, SUVmax

## Abstract

**Introduction:**

^177^Lu-DOTATATE-based peptide receptor radionuclide therapy (PRRT) is a promising therapy for metastatic and/or inoperable pheochromocytoma and paraganglioma (PPGL). We aim to evaluate the efficacy and safety of and identify predictors of response to ^177^Lu-DOTATATE therapy in metastatic and/or inoperable PPGL.

**Methods:**

This retrospective study involved 15 patients of metastatic or unresectable PPGL, who received ^177^Lu-DOTATATE PRRT therapy. Clinical, biochemical (plasma-free normetanephrine), and radiological (anatomical and functional) responses were compared before and after the last therapy.

**Results:**

A total of 15 patients (4 PCC, 4 sPGL, 5 HNPGL, 1 PCC + sPGL, 1 HNPGL + sPGL) were included. The median duration of follow up was 27 (range: 11–62) months from the start of PRRT. Based on the RECIST (1.1) criteria, progressive disease was seen in three (20%), stable disease in eight (53%), partial response in one (7%), and minor response in three (20%) and controlled disease in 12 (80%). On linear regression analysis the presence of PGL (*P*= 0.044) and baseline SUV_max_ >21 (*P* < 0.0001) were significant positive predictors of early response to PRRT. Encouraging safety profiles were noted with no long term nephrotoxicity and hematotoxicity.

**Conclusion:**

^177^Lu-DOTATATE therapy is an effective and safe modality of treatment for patients with metastatic/inoperable PPGL. Although it is not prudent to withhold PRRT in metastatic PPGL with baseline SUV_max_ < 21, baseline SUV_max_ >21 can be used to predict early response to PRRT.

## Introduction

Pheochromocytomas (PCC)–paragangliomas (PGL) (PPGL) are rare tumors of neural crest origin with malignant potential. The prevalence of metastasis ranges from 2–13% in PCC to 2.4–50% in PGL (1, 2). In patients with unresectable, locally advanced or metastatic PPGL, symptomatic or progressive disease is usually treated with chemotherapy, radionuclide therapy (^131^I-metaiodobenzylguanidine (^131^I-MIBG) and peptide receptor radionuclide therapy (PRRT)), external radiotherapy, radiofrequency ablation therapy or tyrosine kinase inhibitors (3, 4). There is no head to head trials that compare the superiority of one modality of therapy over the other.

^177^Lu-tetra-aza-cyclo-dodecanetetraacetic acid–DPhe1-Tyr3-octreotate (DOTATATE) has shown favorable efficacy in controlling symptoms and tumor progression in most of the previous studies (5, 6). A recently published meta-analysis has reported good efficacy (disease control rate: 80% (95% CI: 77–89%)) with an encouraging safety profile (7). But availability, cost, and potential adverse effects limit the use of PRRT.

Identifying predictors of early response to PRRT may help with appropriate patient selection. So far, the only recognized predictor of response to PRRT in PPGL patients is the Ki-67 labeling index (6). Various studies of PRRT in neuroendocrine tumors (NET) have found baseline SUV_max_ as an important predictor of response to PRRT (8, 9). In this study, we have evaluated the efficacy, safety, and predictors of response of ^177^Lu-DOTATATE therapy in metastatic/inoperable PPGL.

## Materials and methods

Retrospective evaluation of consecutive metastatic (*n* = 10) or unresectable (*n* = 5) PPGL patients who received ^177^Lu-DOTATATE therapy (registered at KEM Hospital, Mumbai, India) between January 2010 and December 2019 and followed up for at least 6 months after the first dose of therapy. The study was approved by Institutional Ethical Committee (IEC-II) of Seth G.S. Medical College and KEM Hospital (EC/OA-171/2018) with the waiver of consent. PPGL was diagnosed on basis of histopathology; and in unresectable or metastatic disease, on biochemistry and imaging. All other details including symptomatology, biochemistry, imaging (contrast-enhanced CT (CECT), ^68^Ga-DOTATATE PET/CT, and ^131^I-MIBG scintigraphy), the dose of radionuclide and adverse effects were reviewed. Plasma-free metanephrines (metanephrine (PFMN); normetanephrine (PFNMN)), CECT, and ^131^I-MIBG were done as described previously (2, 10, 11). Genotype was available for few patients (*n* = 5) and was done as described previously (12).

^68^Ga-DOTATATE PECT/CT was performed by administering 111–185 MBq (3–5 mCi) of ^68^Ga-DOTATATE intravenously and obtaining non-contrast-enhanced PET/CT 60 min later on Philips Gemini TF PET/CT (Philips Health Care, USA). Image reconstruction was done using the row action maximum likelihood algorithm (RAMLA). SUV_max_ was calculated by selecting lesions of maximum tracer uptake and more than 1 cm (up to a maximum of five lesions per organ) as regions of interest (ROIs) (9). SUV_max_, SUV_max_ tumor/liver (T/L), or tumor/spleen (T/S) were calculated in ^68^Ga-DOTATATE PET/CT by using the inbuilt software attached to PET workstation. For patients having one lesion, single lesion SUV_max_ and in patients having more than one lesion (more than 1 cm) mean SUV_max_ was calculated.

## PRRT: administration protocol

Eligibility for ^177^Lu-DOTATATE therapy was a high level (Krenning score more than II) and low ^131^I-MIBG uptake. Preparation of ^177^Lu-DOTATATE (by in house generator) and labeling of octreotate with ^177^Lu was done at Radiation Medicine Centre (RMC), Mumbai. Baseline parameters (clinical, biochemical, hematological, functional renal scintigraphy) were noted. PRRT was deferred for patients with one or more cytopenias (hemoglobin <9 g/dL, total leukocyte count <4000/µL or platelet count <100,000/µL), Karnofsky performance status (KFS) less than 60% or the Eastern Cooperative Oncology Group (ECOG) performance status score more than two (13). Patients were premedicated with antihistamines, ondansetron, and positively charged renoprotective amino (l-lysin, l-arginine, etc.) infusion. ^177^Lu-DOTATATE was administered as a slow i.v. infusion over 30–45 min (150–200 mCi/cycle) and observed for a day. A maximum of six cycles was given with a minimum interval of three months was maintained between 2 consecutive cycles.

## Assessment of efficacy

Efficacy was assessed based on clinical (compressive or catecholaminergic features, change in antihypertensive medications), plasma-free metanephrines (PFMN and PFNMN), CECT, and SSTR response. Plasma concentrations of free metanephrines (single value) before the commencement of therapy and after the last PRRT cycle were compared. For anatomical imaging CECT was used and the CECT response (gold standard) was based on RECIST version 1.1 (target lesion). Complete response (CR) was defined as the disappearance of all target lesions plus reduction of the short axis of pathologic lymph nodes to <1 cm, partial response (PR) as at least 30% decrease in the sum of the longest diameters of target lesions (relative to baseline sum), minor response (MR) as smaller decrements in size not meeting the criteria of PR (10–30% decrease in maximum diameters of target lesions), stable disease (SD) as neither MR nor progressive disease (PD) and PD as at least 20% increase (≥5 mm absolute increase) in the sum of longest diameters of target lesions (relative to smallest sum) or appearance of new lesions (14, 15). Controlled disease (CD) was defined as a combination of all the responses except PD (SD + PR + MR). CD was calculated to make the data more comparable to previously published literature (5, 7).

SSTR response was based on ^68^Ga-DOTATATE PET/CT (defined as partial response (PR): reduction in intensity by one Krenning score in at least one tumor site, complete response (CR): total disappearance of abnormal uptake of previous avid lesions) or progressive disease (PD): increase in intensity or extent of previous abnormal uptake, or development of new avid lesions) (5, 16, 17, 18).

## Statistical analysis

Statistical analysis were done using SPSS (version 23, IBM) and MedCalc Ink (version 19.1.6). Categorical variables were expressed in actual numbers and percentages. Continuous variables were expressed as mean ± s.d. or median and range as appropriate. Continuous variables between the two groups were compared using the Mann–Whitney *U*-test whereas categorical variables were compared using Fischer’s exact *t*-test. Progression-free survival (PFS) and overall survival (OS) were determined by using Kaplan–Meier analysis and PFS was compared between the groups using the log-rank test. We used receiver operating characteristic (ROC) curve analysis and area under the curve (AUC) to determine the optimal cut-off of baseline SUV_max_, which predicts response to PRRT. Linear regression analysis was performed to identify predictors of response to PRRT. Kappa coefficient was calculated to compare the agreement between RECIST 1.1 and change in SUV_max_ of <15% for diagnosing PD and CD. *P*-value < 0.05 was considered as statistically significant.

## Results

A total of 15 patients ((7 males); (4 PCC, 4 sPGL, 5 HNPGL, 1 PCC + sPGL, 1 HNPGL + sPGL)) were included. The mean age at the start of PRRT was 32.5 ± 13.9 years. All PCC and sPGL except one sPGL were normetanephrine-secreting (median: 677 pg/mL (range: 216–3296)). All HNPGL were non-secretory. Three patients had a concomitant pancreatic neuroendocrine tumor (PNET) of whom two were genetically proven to have von Hippel Lindau syndrome. The indication for PRRT was metastasis (8) and inoperability (7). A median of three cycles (1–6) was administered with median cumulative radioactivity of 28 GBq (19–40 GBq). Other tumor characteristics are summarized in Table 1. None of the patients in our cohort have received somatostatin analog therapy.

**Table 1:** Baseline characteristics of the study cohort.

Case no.	Sex	Age at start of therapy	Primary tumor	Indication for PRRT	Site of metastasis	Secretory status	Number of PRRT cycles	Cumulative dose of PRRT (GBq)	Follow up (in months)	Baseline mean^a^ SUV_max_ (Krenning score)	Previous therapy		Mutation/amino acid
											Surgery	EBRT	
1	M	14	PCC + PNET	Progressive, inoperable	NA	S	5	25	32	5.7 (II)	+	−	*VHL* (Exon:3)/p.(Arg167Trp
2	M	27	PCC + sPGL + PNET	Progressive, metastasis (metachronous)	Liver	S	3	10	21	25.5 (IV)	+	−	*VHL* (Exon:1)/ p.(Tyr98Ser)
3	F	18	sPGL	Progressive, metastasis (metachronous)	Skeletal, lung, LN	S	6	37	62	28.5 (IV)	−	−	*SDHB* (Exon:4)/p.Gly96Asp
4	M	38	HNPGL + sPGL	Inoperable	NA	S	6	40	35	106 (IV)	−	−	*SDHD* (Exon:4)/p.Ser8LysfsTer6
5	M	59	PCC	Progressive, metastasis (metachronous)	Lung, liver, LB	S	3	19	17	12.8 (III)	+	−	Negative
6	F	22	PCC + PNET	Progressive inoperable	NA	S	3	19	27	40 (IV)	+	−	ND
7	F	39	sPGL	Progressive, metastasis (metachronous)	LN, LB	S	2	11	54	7.6 (II)	+	−	ND
8	F	37	PCC	Progressive, metastasis (metachronous)	Skeletal, liver	S	4	28	15	17.4 (III)	+	−	ND
9	M	49	sPGL (bladder)	Progressive, metastasis (synchronous)	Skeletal	S	2	15	11	35.5 (IV)	+	−	ND
10	M	44	sPGL	Inoperable + metastasis (synchronous)	Skeletal	NS	1	6	11	61.5 (IV)	+	+	ND
11	M	39	HNPGL	Inoperable + metastasis (synchronous)	Skeletal, liver, lymph node	NS	5	30	52	41.7 (IV)	−	+	ND
12	F	18	HNPGL	Inoperable + metastasis (synchronous)	Lung	NS	6	35	36	−	−	−	ND
13	F	41	HNPGL	Inoperable	NA	NS	6	40	34	138 (IV)	+	−	ND
14	F	25	HNPGL	Inoperable	NA	NS	5	34	26	254 (IV)	+	−	ND
15	F	42	HNPGL	Inoperable + metastasis (synchronous)	Skeletal	NS	5	32	27	57 (IV)	−	+	ND

^a^For a patient having one lesion, single lesion SUV_max_, and more than one lesion mean SUV_max_ were calculated.

EBRT, external beam radiotherapy; F, female; HNPGL, head and neck paraganglioma; LB, local bed; LN, lymph node; M, male; NA, not applicable; ND, not done; NS, non-secretory; PCC, pheochromocytoma; PNET, pancreatic neuroendocrine tumor; S, secretory; sPGL, sympathetic paraganglioma.

**Figure 1:**
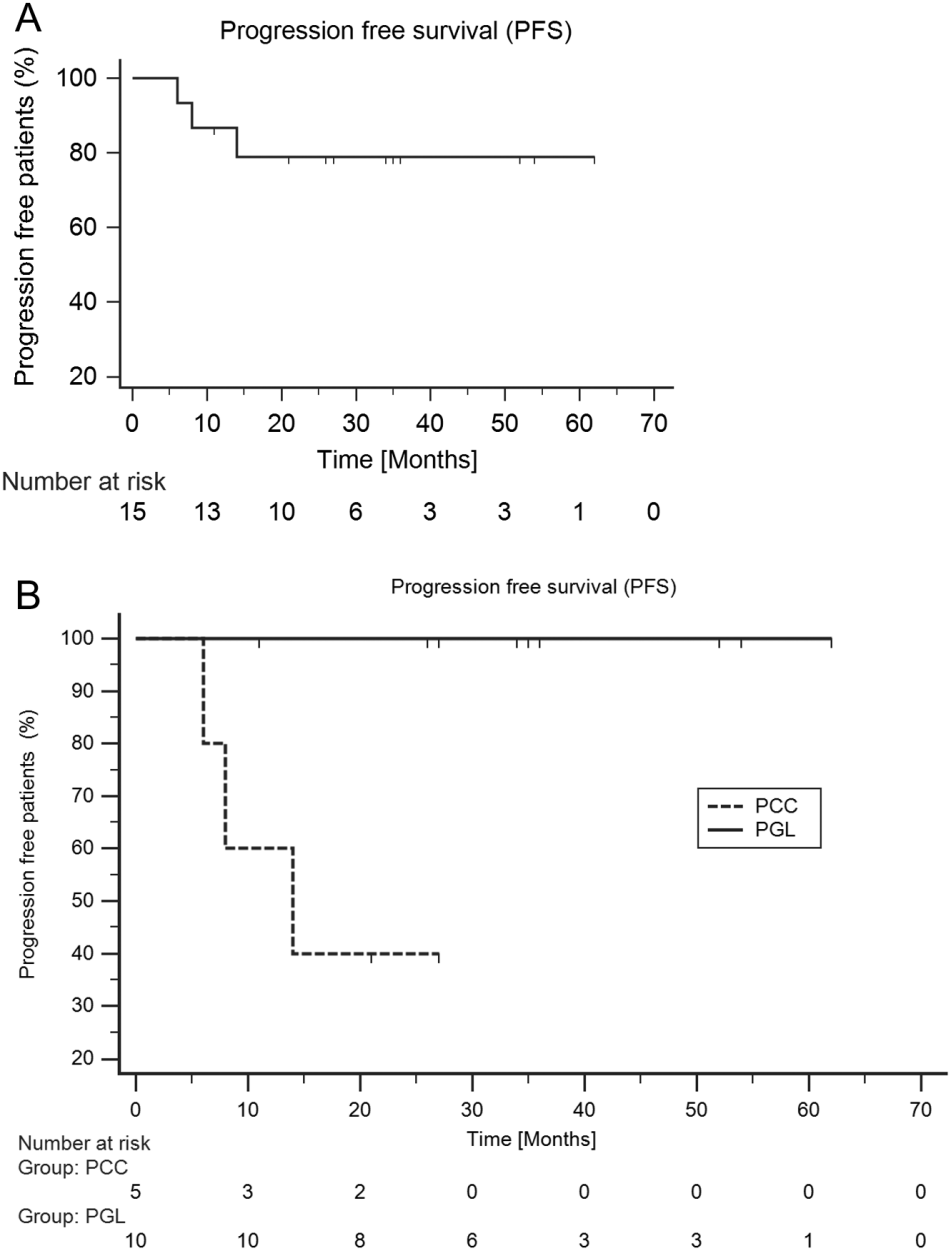
(A) Kaplan–Meier plots for PFS of the overall primary lesion (HNPGL + Spgl + PCC), (B) Kaplan–Meier plots (log-rank test) to compare PFS among PCC vs PGL.

The median duration of follow up was 27 months (range: 11–62) from the start of PRRT. The overall survival was 100% whereas median PFS was not reached (Fig. 1A). None of the HNPGL patients had PD. Median PFS was 14 months in PCC whereas median PFS was not achieved in HNPGL (Fig. 1B).

Based on RECIST (1.1) criteria, PD was seen in three (20%), SD in seven (47%), PR in one (7%), and MR in four (27%) and CD in 12 (80%).

All three patients with PD had PCC and PCC were significantly more frequent in them than those with CD (p=0.04). In patients who had PD even after three cycles of PRRT, other modalities (Actinium-225(^225^Ac)-based PRRT (*n* = 2) or high dose ^131^I-MIBG therapy (*n* = 1)) were used on compassionate basis whereas patients who had SD or PR were offered to complete the course of six cycles.

**Figure 2:**
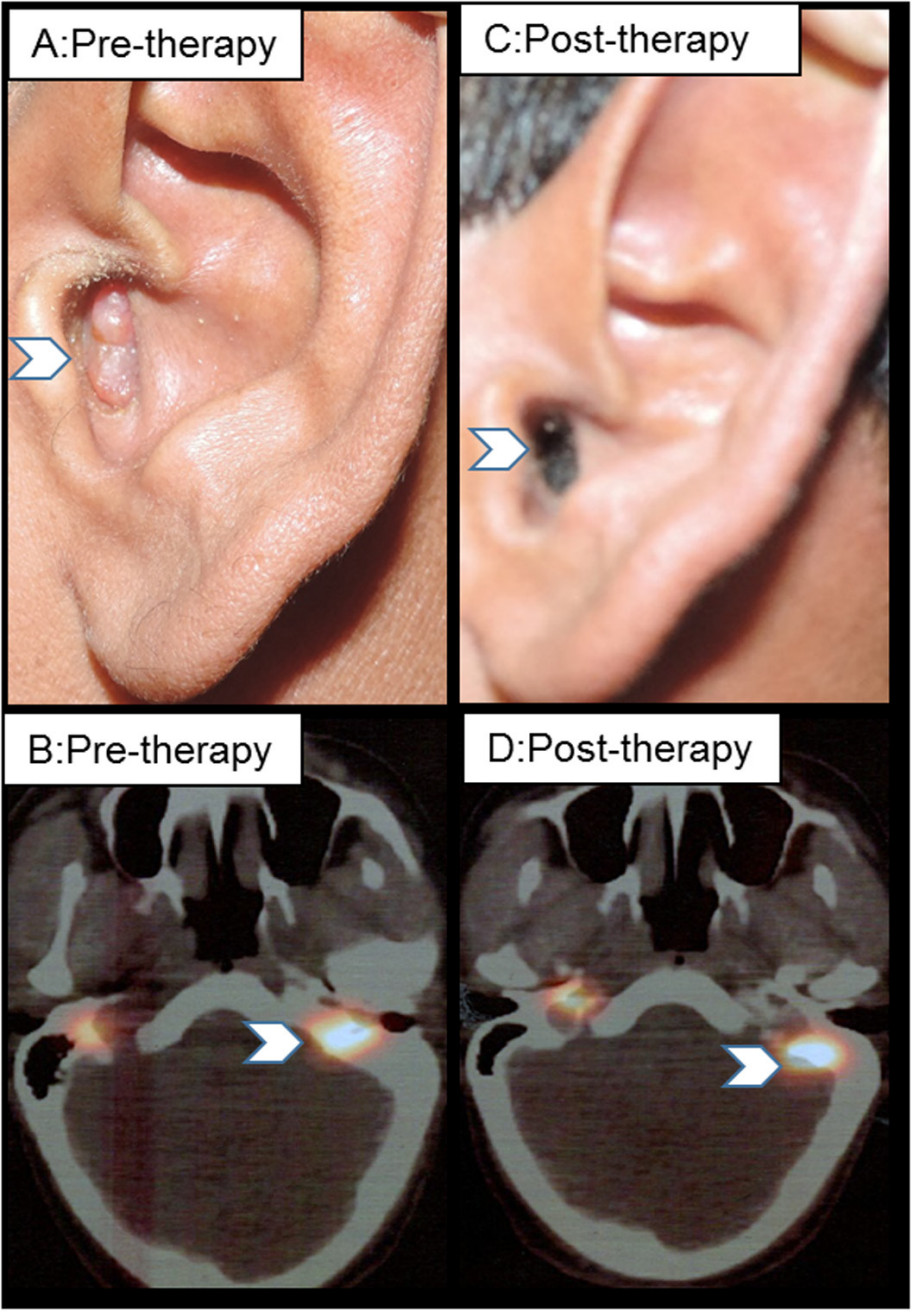
Response in SDH-D positive (case 4) unresectable HNPGL following six cycles of PRRT therapy ((A, B) pretherapy mean SUV_max_: 104 and (C, D) posttherapy mean SUV_max_: 24, respectively).

Worsening of symptoms was observed in only two patients with PD (13%) whereas the remaining (12 controlled; 1PD) had improvement or no change in symptoms. A patient (case 4), had significant tinnitus and the fleshy tumor was visible in the left external auditory canal (EAC), however, after six cycles of PRRT therapy, marked improvement in the tinnitus and disappearance of the EAC lesion (Fig. 2).

The defined daily dose (DDD) of anti-hypertensives decreased in six of nine (66%) patients while increased in the remaining three (PD). As all the nine secretory PPGL were normetanephrine-secreting, change in PNFMN after PRRT was calculated. After the last cycle of PRRT, five (56%) patients had decreased PFNMN level, with a percentage fall of >50, 25–50, and 10–25% in two, one, and two patients, respectively. Four patients had increased PFNMN level after PRRT of whom three had PD and one had SD (Table 2).

**Table 2:** Response evaluation in all patients after ^177^Lu-PRRT therapy.

Case no.	Symptoms	Anti-hypertensive (before)	Anti-hypertensive (after)	ΔDDD of antihypertensives (%)	ΔPFNMN (%)	ΔMean diameter^a^ (%)	Best response (RECIST 1.1)	SSTR-Response	∆STR-R^b^ SUV_max_ (%) on SSTR image
1	Stable	Amlodipine: 2.5 mg	Amlodipine: 12.5 mg	400	520	200	PD	PD	52.6
2	Stable	Prazosin: 7.5 mg, Amlodipine: 5 mg	Prazosin: 7.5 mg	−40	16.6	−7.2	SD	SD	−71.8
3	Improved	Prazosin: 5 mg, Metoprolol: 50 mg	Prazosin: 2.5 mg, Metoprolol: 25 mg	−50	−69.4	−16.3	MR	PR	−62.1
4	Improved	Prazosin: 20 mg, Atenolol: 50 mg	0	−100	−14.8	−22	MR	PR	−76.7
5	Worsened	Amlodipine: 10 mg	Prazosin: 30 mg, Metoprolo: 200 mg	266	1333	34.5	PD	PD	17.1
6	Improved	Prazosin: 20 mg Atenolol: 25 mg	0	−100	−17.3	−9.17	SD	SD	−50
7	Improved	Prazosin: 10 mg	0	−100	−89.7	−6	SD	SD	−18.3
8	Worsened	Amlodipine: 10 mg	Amlodipine: 10 mg, Prazosin: 15 mg	150	168	49.9	PD	PD	22.9
9	Improved	Prazosin: 30 mg, Amlodipine: 10 mg	Prazosin: 15 mg	−62.5	−45.2	−2.8	SD	SD	−41.5
10	Improved	NHTN	–	0	NA	NA	SD	SD	NA
11	Stable	NHTN	–	0	NA	−2.47	SD	SD	209
12	Improved	NHTN	–	0	NA	−8	SD	SD	–
13	Improved	NHTN	–	0	NA	−20	MR	PR	−75.3
14	Improved	NHTN	–	0	NA	−40	PR	PR	−76.3
15	Stable	NHTN	–	0	NA	−17	MR	SD	−1.7

^a^Change in the longest diameter for non-nodal lesions and short axis for lymph nodes; ^b^For a patient having one lesion, single lesion SUVmax, and more than one lesion mean SUV_max_ were calculated.

DDD, defined daily dose; MR, minor response; NA, not available; NHTN, Normotensive; PD, progressive disease; PFNMN, plasma free normetanephrine; PR, partial response; SD, stable disease.

**Figure 3:**
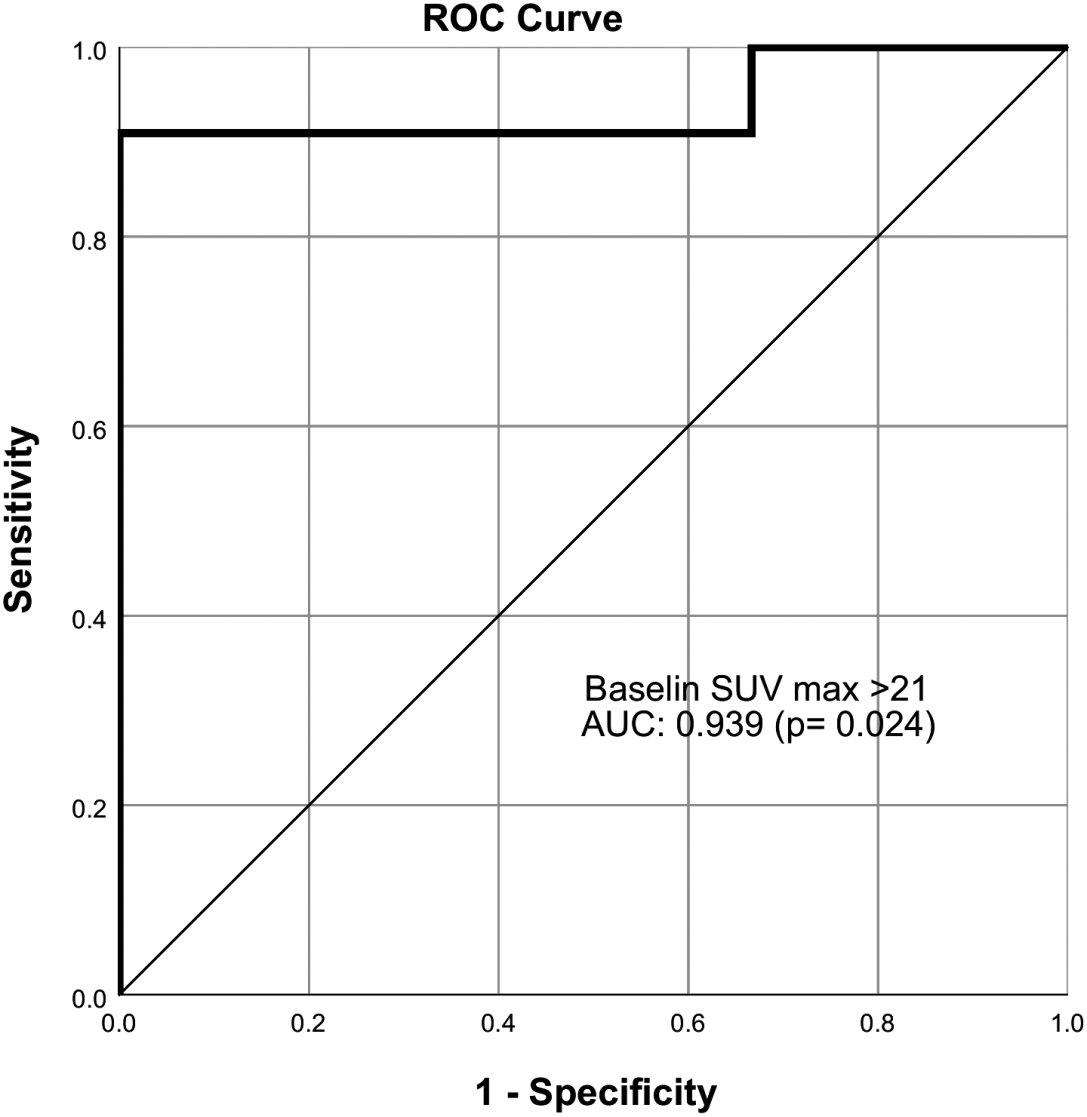
Receiver operating characteristic (ROC) curve of baseline SUV_max_.

The baseline mean SUV_max_ on ^68^Ga-DOTATATE was numerically higher in the CD compared to PD (72.4 ± 70.9 vs 11.9 ± 5.8, *P* = 0.179) but statistically insignificant albeit with a cut-off of SUV_max_ of >21 the difference was clinically significant (nine out of ten; 90% of CD; *P* = 0.004). In the ROC curve analysis done which included 14 patients, the baseline SUV_max_ significantly predicted response (CD) to PRRT with the area under the curve (AUC) of 0.939 (*P* = 0.024) (Fig. 3). SUV_max_ of >21 had a sensitivity of 0.91 (95% CI: 0.80–1.00) and specificity of 1.0 (95% CI: 0.29–1.00) to predict response to PRRT. The PFS was apparently longer in patients with baseline SUV_max_ of >21 compared to those with < 21 (35 (11–62) months vs 11 (6–54) months).

**Figure 4:**
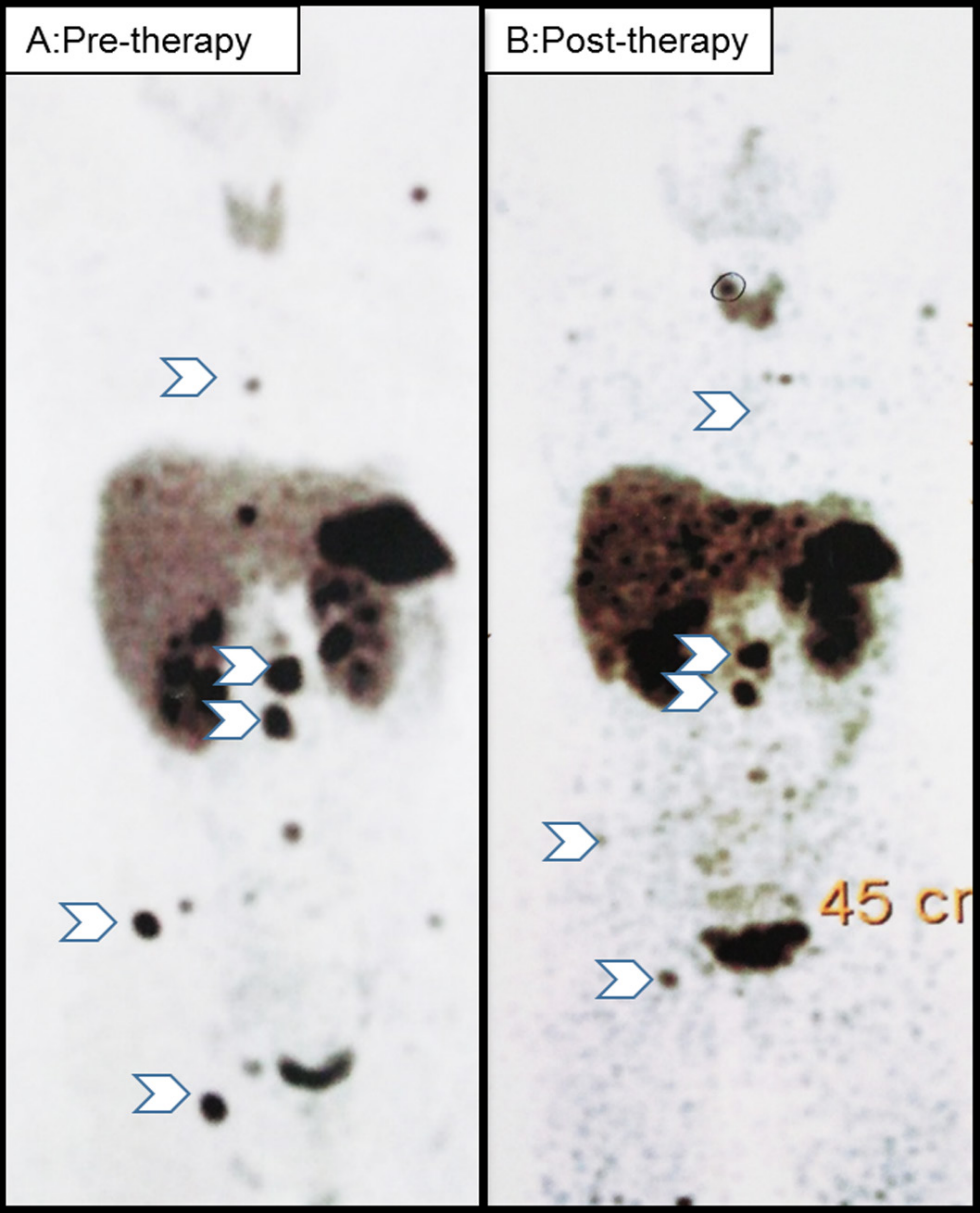
Showing response in SDH-B positive (case 4), metachronous metastatic sPGL (pre-therapy (A) and post-therapy (B) mean SUV_max_ 28.5 and 10.5, respectively).

Baseline mean SUV_max_ was significantly higher among HNPGL patients than those with PCC or sPGL (119 ± 84.5 vs 26.1 ± 17.8, *P* = 0.006). On linear regression analysis baseline SUV_max_ >21 (*r*^2^ = 0.682, *P* < 0.0001) and PGL (*r*^2^ = 0.783, *P* = 0.04) were the significant positive predictors. Change in SUV_max_ before and after PRRT was available for 13 patients. Nine (69%) patients had decreased and four (31%) had increased mean SUV_max_ (17–210%). Among the latter four, three had PD while one had SD on RECIST 1.1. An increase in SUVmax by >15% was observed in all the three patients with PD whereas eight of 10 patients with CD had a decrease in SUV_max_ by >15% (Table 3). Change in SUV_max_ of >15% after PRRT had good agreement with the diagnosis of PD and CD based on RECIST 1.1, as shown in Fig. 4 (case 3) a metastatic sPGL, after six cycles of PRRT, −62 % change in mean SUV_max_ and CD based on RECIST 1.1.

**Table 3:** Comparison between progressive disease and controlled disease.

**Parameters**	Progressive disease (PD)	Controlled disease (PR + SD + MR)	*P*-value (95% CI)
Mean age (years)	36.6 ± 22.5	42 ± 10.8	0.544
Tumor type	PCC: 3	PCC (1), sPGL(4), HNPGL(5), sPGL + HNPGL: 1, sPGL + PCC: 1	0.004^a^
Hypertension, *n* (%)	3 (100)	7 (58.3)	0.20
Pre-therapy PFNMN (mean ± s.d.)	749 ± 1097	1321 ± 1369	0.93
Change in DDD (%), *n* = 3 + 7 (mean ± s.d.)	+207 ± 132	−50.2 ± 43.6	0.001^a^
Change in PFNMN (mean ±s.d.)	+673 ± 597	−11.1 ± 31.4	0.000^a^
No of PRRT cycle ( mean ± s.d.)	3.5 ± 2.08	4.1 ± 1.8	0.63
Mean dose of Lu (GBq) ( mean ± s.d.)	23.6 ± 14.4	26.2 ± 1.9	0.49
Pre-therapy ^68^Ga-DOTATATE SUV_max_ (mean), *n* = 13 (mean ± s.d.)	12.4 ± 6.0	72.4 ± 70.9	0.18
Baseline SUV_max_>21, *n* = 14, *n* (%)	0 (*n* = 3)	90 (9/10)	0.004a
Baseline mean (T/L) ( mean ± s.d.)	1.2 ± 0.6	9.1 ± 9.4	0.18
Baseline mean (T/S) (mean ± s.d.)	0.49 ± 0.28	3.3 ± 4.2	0.28
Reduction of mean SUV_max_ (tumor) more than 15% (*n* = 13)	0/3 (0)	8/10 (80)	0.022^a^
Reduction mean SUV_max_ (T/L) > 15%, *n* (%)	0/3 (0)	5/8 (63)	0.07
Reduction mean SUV_max_ (T/S) > 15%, *n* (%)	2/3 (33)	4/8 (50)	0.63

^a^*P*-value <0.05.

DDD, defined daily dose; HNPGL, head and neck paraganglioma; MR, minor response; PCC, pheochromocytoma; PD, progressive disease; PFNMN, plasma-free normetanephrine; PR, partial response; SD, stable disease; sPGL, sympathetic paraganglioma; SUV_max_, standard uptake value maximum; T/L, tumor/liver; T/S, tumor/spleen.

In patients with associated PNET (*n* = 3), the mean SUV_max_ of PNET decreased (57 ± 33 vs 20 ± 9.4, *P* = 0.137) on follow up imaging, despite progressive PPGL disease in one patient.

The most common adverse effects observed were nausea-vomiting (*n* = 3, 20%) and weight loss (*n* = 2, 13%). One patient had isolated grade 2 thrombocytopenia and another had combined anemia and thrombocytopenia which recovered in 2 and 4 weeks, respectively. None of the patients had transient or permanent nephrotoxicity. Mean glomerular filtration rate and renal plasma flow were 121 ± 19 and 505 ± 99.4 mL/min following the last cycle of PRRT therapy. None had a catecholaminergic crisis during or after therapy. One patient with childhood-onset of sPGL developed bilateral avascular necrosis of hip after 26 months of PRRT.

## Discussion

Our study reiterates the efficacy of PRRT in controlling metastatic/inoperable PPGL. Using the RECIST 1.1 criteria, none of our patients had a complete response as reported by most of the previous studies whereas three (20%) patients had a progressive disease which is also in agreement with the existing literature (14–16%). Twelve patients had CD yielding a disease control rate of 80% which is comparable to that reported in a recent meta-analysis (84% (95% CI: 77%‐89%)). Objective response rate (morphological reduction) including CR, PR and MR in our study (33% (5/15)) was also comparable to that reported in the meta-analysis (25% (95% CI: 19%‐32%)). However, the PR rate was relatively lower in our cohort (6.7%) than that reported in previous studies (7–29%). This may be due to the limited use of additional treatments in our cohort than that in most of the previous studies. This feature may be the strength of our study as the effect of PRRT is least likely to be confounded.

Clinical improvement is seen in a majority of patients subjected to PRRT, with worsening of symptoms being documented in only two patients (13%) who had PD. This is in accordance with the existing literature where the worsening of symptoms has been reported in 11–12% of patients (5, 19). Thus, clinical features can be useful markers of response to PRRT. Four (44%) of the nine patients had increased PFNMN level after PRRT which concurred with progressive disease in three patients whereas in the other with stable disease, the increase in PFNMN was milder (<20%). Thus, PFNMN may also be a useful marker to assess response to PRRT in patients with secretory PPGL.

We demonstrated that baseline mean SUV_max_ on ^68^Ga-DOTATATE PET/CT may be used as a parameter to predict the response to therapy. The prediction accuracy was high which resulted in significant prediction despite smaller sample size. The utility of mean SUV_max_ on 68Ga-DOTATATE to predict response to PRRT has been described in the context of pancreatic and bowel NET (8, 9). However, the available literature on PRRT in PPGL, especially regarding the predictors of response to PRRT is sparse. Baseline mean SUV_max_ of 21 on ^68^Ga-DOTATATE significantly predicted response to subsequent PRRT in our study. Patients with baseline mean SUV_max_ of >21 also tended to have longer PFS. Similar SUV_max_ cut-offs (13–26.3) have been obtained to predict response to PRRT among NET patients (9). Better response to PRRT in NET with higher SUVmax is expected as they provide more receptors for binding of the radiopharmaceutical allowing higher radioactivity in the lesion. However, a recent study has shown better histological differentiation of tumors with higher SUV_max_ (20) which may also contribute to the better response of NET with higher SUV_max_. Hence, the majority of the patients with CD could be predicted to have a favorable response by baseline SUV_max_ of >21.

Anatomical criteria such as RECIST 1.1 are a quantitative measure to define response to PRRT and is considered the gold standard criteria. However, there are no functional imaging-based quantitative criteria to assess response to PRRT. In our study the diagnosis of PD and CD based on reduction in SUV_max_ by 15% after PRRT had a substantial agreement (k = 0.64, = 0.012) with RECIST 1.1 criteria. Hence, SUV_max_ may be used as a quantitative measure to assess response to PRRT.

Interestingly, PCC was a significant negative predictor of response to PRRT. Similarly, Nastos* et al.* have reported better response to PRRT in PGL than in PCC (3). The poorer response of PCC than PGL may be due to less expression of SSTR in the former as noted in our study (20.2 ± 13.1 vs 122 ± 97.2, *P* = 0.04). On the other hand, the poor response may be due to their more dedifferentiated nature as suggested by poor uptake of ^131^I-MIBG as well as ^68^Ga-DOTATATE at baseline. However, considering the limited number of therapeutic options available for metastatic PCC, especially for those with low MIBG uptake, the limited data from our study does not preclude the use of PRRT in them.

HNPGL had a trend for better disease control and longer PFS which has also been reported in a recent study (21). SUV_max_ was significantly higher among HNPGL patients which might have resulted in the tendency for better response. However, a higher proportion of nonmetastatic disease (50–65%) among HNPGL than PCC + sPGL (22–85%) (21), or the inherent nature of metastatic HNPGL to progress slowly might have also contributed for the better response (1).

In addition to good efficacy, ^177^Lu-based therapy has been reported to have an encouraging safety profile. Apart from minor gastrointestinal side effects, only two of our patients developed transient grade I thrombocytopenia, with no other serious toxicity. In the Netter-1 trial including 116 patients with mid-gut NET who received PRRT, grade 3 or 4 neutropenia, thrombocytopenia, and lymphopenia occurred in 1, 2, and 9%, respectively, whereas none of them developed nephrotoxicity and myelodysplastic syndrome (MDS) (21). Although catecholaminergic crisis and tumor lysis syndrome have been reported in PPGL patients receiving PRRT (22, 23), none of our patients developed these adverse effects. However, most of our secretory PPGLs had received adequate alpha blockade before being subjected to therapy which might have masked the potential for catecholaminergic crisis.

The number of PRRT cycles was variable (one to six) in our study; two patients with sPGL received only one to two cycles (cases 9 and 10). However, fewer cycles in these patients are unlikely to underestimate the response to PRRT as both these patients had SD despite only one to two cycles. Notably, one of them also achieved more than 40% reduction in DDD a well as PFNMN.

The major limitations of our study are the small sample size and retrospective nature; thus MIB1-index, chromogranin A, and 68Ga-DOTATATE-PET/CT values were not available at several time points of follow up. Another major limitation of the study is lack of genetic data in many patients. It is possible that a trend for better response among patients with HNPGL than those with sPGL might be due to association of the former with mutations in *SDHD* rather than in *SDHB*. However, such an analysis could not be performed due to limited genetic data. Also, it was not possible to ascertain with certainty whether the SD was the effect of PRRT or the natural course of the disease. However, in a few patients with SD decreasing trend of SUV_max_ was noted after PRRT which indirectly demonstrates the responsive nature of the disease. Hence, prospective, randomized, controlled studies with larger patient numbers are warranted.

## Conclusion

^177^Lu-DOTATATE therapy is an effective and safe modality of treatment for patients with metastatic/inoperable PPGL. Baseline SUV_max_ more than 21 on ^68^Ga-DOTATATE positively predicts early response to ^177^Lu-DOTATATE therapy. Although it is not prudent to withhold ^177^Lu-DOTATATE therapy in metastatic PPGL with baseline SUV_max_ < 21, baseline SUV_max_ >21 can be used to predict early response to ^177^Lu-DOTATATE therapy. We also found that the change in SUV_max_ on follow-up imaging may be a useful parameter, in addition to clinical, biochemical, and radiological parameters, to monitor the response to ^177^Lu-DOTATATE therapy. However, the study findings need confirmation in larger, prospective cohorts.

## Declaration of interest

The authors declare that there is no conflict of interest that could be perceived as prejudicing the impartiality of the research reported.

## Funding

This research did not receive any specific grant from any funding agency in the public, commercial or not-for-profit sector.
